# The effect of supplemental oxygen and continuous positive airway pressure withdrawal on endocan levels

**DOI:** 10.1007/s11325-024-03120-2

**Published:** 2024-09-06

**Authors:** Chris D Turnbull, John R Stradling, Nayia Petousi, Philippe Lassalle

**Affiliations:** 1 Oxford Centre for Respiratory Medicine, Oxford University Hospitals NHS, Foundation Trust, Oxford, UK; 2 Nuffield Department of Medicine, University of Oxford, Oxford, UK; 3 NIHR Oxford Biomedical Research Centre, University of Oxford, Oxford, UK; 4 INSERM U1019, Institut Pasteur de Lille, Lille, France

**Keywords:** Obstructive sleep apnoea, Continuous positive airway pressure, Cardiovascular disease, Intermittent hypoxia, Endothelial dysfunction, Endocan

## Abstract

**Purpose:**

Endocan is a biomarker of endothelial dysfunction, which is a precursor to cardiovascular disease. Obstructive sleep apnoea (OSA) is associated with elevated endocan levels but the effects of treatment on endocan levels in OSA are not fully established. We aimed to determine whether endocan levels could be detected by immunoassay and to determine the effect of supplemental oxygen during continuous positive airway pressure (CPAP) withdrawal on circulating endocan levels.

**Methods:**

We conducted an exploratory analysis from a randomised controlled crossover study which included participants with OSA. Participants stopped their CPAP therapy and were randomised to receive either supplemental oxygen or sham for 14 nights before crossing over. Supplemental oxygen blocked the rise in blood pressure seen in the sham group. We analysed plasma endocan levels by immunoassay at baseline and after 14 nights of intervention in both groups.

**Results:**

Twenty-five participants were included, with a total of 100 samples. Endocan levels were detectable at all time points in 22 participants (88%), and in 93 (93%) samples. Supplemental oxygen had no effect on endocan levels compared to sham (+ 0.52 ng/ml, 95%CI -0.21 to + 1.25, *p* = 0.16), and there was no significant difference in endocan levels from baseline to follow-up in either the sham (-0.30 ng/ml, 95%CI -0.89 to + 0.30, *p* = 0.31) or supplemental oxygen (+ 0.22 ng/ml, 95%CI 0.00 to + 0.44, *p* = 0.05) arm.

**Conclusions:**

We have shown that endocan levels are detectable before and after CPAP withdrawal. However, we found no effect of supplemental oxygen following CPAP withdrawal on circulating endocan levels.

**Trial registration and date:**

ISRCTN 17,987,510 19/02/2015.

## Introduction

Obstructive sleep apnoea (OSA) is associated with endothelial dysfunction which is a precursor to cardiovascular disease, and intermittent hypoxia is thought to be central to the development of both endothelial dysfunction and cardiovascular disease in OSA [1]. Endocan is a proteoglycan that is produced by vascular endothelium and the blood endocan variations are associated with the occurrence of pneumonia and acute respiratory distress syndrome [2]. Endocan is a biomarker of endothelial dysfunction which is expressed in response to intermittent hypoxia and is hypothesised to be involved in the pathogenesis of cardiovascular disease in OSA [3]. A recent meta-analysis proposed endocan as a diagnostic and prognostic biomarker for OSA [4]. An observational study showed increased endocan levels in individuals with moderate to severe OSA compared to controls, and increased endocan levels were reduced by continuous positive airway pressure (CPAP) treatment but remained higher than controls [5]. However, in contrast we previously showed that two weeks of CPAP treatment withdrawal, leading to recurrence of OSA, had no effect on endocan levels when measured using a commercially available enzyme-linked immunosorbent assay kit [6]. Our previous analysis was limited as endocan levels were below the lower limit of detection in over 50% of our participants. In this present exploratory study, we sought to determine if using a more sensitive immunoassay [7], improved the detection of circulating plasma endocan levels during CPAP withdrawal, and whether supplemental oxygen (which attenuated intermittent hypoxia during CPAP withdrawal) impacted endocan levels.

## Methods

### Study design and participants

Full methodology of the original study is published elsewhere [8]. In brief, we conducted a randomised double-blind crossover study in participants with known moderate to severe OSA already established and effectively treated with CPAP (with good CPAP usage of > 4 h/night). Participants stopped CPAP therapy and received either 14 nights of supplemental oxygen or 14 nights of sham (air), before crossing over with randomised treatment order, and a washout of at least 14 nights back on CPAP therapy between each treatment arm. Supplemental oxygen and sham were delivered via identical concentrators at a flow rate of 5 L/min via either a face-mask or nasal cannulae. Participants and the researchers involved in conducting study visits were blinded to treatment allocation. The primary outcome of the original study was that supplemental oxygen blocked the rise in BP seen following CPAP withdrawal.

### Study outcomes

In this exploratory analysis, the main study outcome was plasma endocan levels. We assessed the number of samples where endocan levels were detectable within the measurable range, and also the effect of supplemental oxygen on endocan levels during CPAP withdrawal. Blood tests for endocan levels were collected at baseline and after two weeks of intervention in both arms. Blood was spun to produce platelet-poor plasma and stored at -80^o^C until study completion, when analysis was performed in one batch. Sample assays were measured at ¼ and ½ dilution using an in vitro diagnostic device based on immunoenzymatic assay (EndoMark H1A, lot 012) to improve the detection of plasma endocan levels (lower and upper limits of quantification 0.6 and 60 ng/mL respectively).

### Statistics

This was an exploratory analysis and as such no formal power calculation was conducted. In another study, CPAP reduced endocan levels from 5.01 ± 3.17 ng/dL to 3.25 ± 2.24 ng/dL. In order not to miss a similar effect of supplemental oxygen on endocan levels to CPAP and assuming a standard deviation of the difference of 2.7, 22 participants would be needed for 80% power with a two-sided significance level of 5%.

Continuous data were assessed for normality and presented as either mean ± standard deviation when normally distributed or median (first quartile, third quartile) when non-normally distributed. Categorical data are presented as number (percentage). The effect of supplemental oxygen on endocan levels was modelled using mixed effect modelling. Mixed effect models included endocan levels as the dependent variable with the treatment effect modelled as the interaction between treatment (supplemental oxygen or sham) and the visit (baseline or follow-up), with adjustments for randomisation order, age, sex, body mass index (BMI), and baseline ODI, and the participant modelled as a random effect. We carried out a sensitivity analysis only including patients with severe obstructive sleep apnoea at diagnosis and explore the correlations (Spearman’s rank correlation) between endocan levels and markers of intermittent hypoxia (ODI, percentage time with saturations less than 90% and the hypoxic burden based on oximetry). The hypoxic burden based on oximetry was measured using online software (https://hypoxicburden.thesiestagroup.com) [9]. Statistics were conducted using SPSS Statistics (Version 28.0, IBM, Armonk, NY, USA) and graphics were created using R studio (Version 2023.06.2 + 561, PBC, Boston, MA, USA).

## Results

We randomised 25 participants with mean age of 62.7 ± 6.8 years, mean BMI of 35.3 ± 6.7 kg/m^2^, and median ODI at diagnosis of 48.1 events/hour (25.3, 70.1). Most participants were male (21 or 84%) with only four (16%) female participants. Four (16%) participants had diabetes mellitus, 15 (60%) were on anti-hypertensive therapy and 7 (28%) were on a statin medication.

### The effect of CPAP withdrawal and supplemental oxygen on intermittent hypoxia

CPAP withdrawal with sham led to a marked return of intermittent hypoxia and an increased hypoxic burden, which were both markedly attenuated but not abolished by supplemental oxygen (Table 1). CPAP had no significant effect on the number of obstructive events as measured by the apnoea hypopnoea index (AHI).

**Table 1:** The effect of CPAP withdrawal with sham and with supplemental oxygen on measures of intermittent hypoxia and the AHI. AHI: Apnoea-Hypopnoea index, ODI: Oxygen desaturations index > 4%, %T90 = percentage of time with oxygen saturations less than 90%, HB_OXI_ = OSA specific hypoxic burden as measured by oximetry, 95%CI = 95% confidence interval. Data are expressed as median (IQR) or by treatment effect (95%CI)

	CPAP withdrawal with sham	CPAP withdrawal with supplemental oxygen	Treatment effect of supplemental oxygen (95%CI)	*p*-value
ODI (events/h)	32.5 (25.6, 47.0)	6.4 (4.0, 14.7)	−23.8 (− 31.0 to − 16.3)	< 0.001
%T90 (%)	14.3 (5.9, 21.2)	2.0 (0.3, 3.9)	−9.8 (− 16.7 to − 4.3)	< 0.001
*HB*_*OXI*_ (%min/h)	109.7 (87.5, 145.2)	32 (16.7, 57)	-73.4 (-92.1 to -54.7)	< 0.001
AHI (events/h)	34.4 (22.7, 44.4)	30.4 (23.6, 42.6)	-3.6 (-10.2 to + 10.1)	0.98

### Endocan detection

A total of 100 samples were collected at four time points (pre- and post both study arms). When endocan levels were assayed at ½ dilution, 92 samples were within the measurement range, with 1 sample above the upper and 7 below the lower limit of detection. When endocan levels were assayed at ¼ dilution, 60 samples were within the measurement range, with 40 below the lower limit of detection. From the 59 samples measured within the range of measurement at both dilutions, all paired values had coefficients of variance of less than 20%. Seven samples had endocan values below the lower limit of detection using both assay dilutions and were ascribed values of 1.20 ng/ml. No samples were above the upper limit by both techniques. Where only one dilution assay gave an endocan level within the range of measurement, this value was recorded. Where paired data was available, the mean of both dilution assays was recorded. In total 93 (93%) of samples had endocan levels within the measurable range and 22 (88%) participants had endocan levels within the measurable range at all time points.

### Effect of supplemental oxygen and CPAP withdrawal on endocan levels

**Fig. 1:**
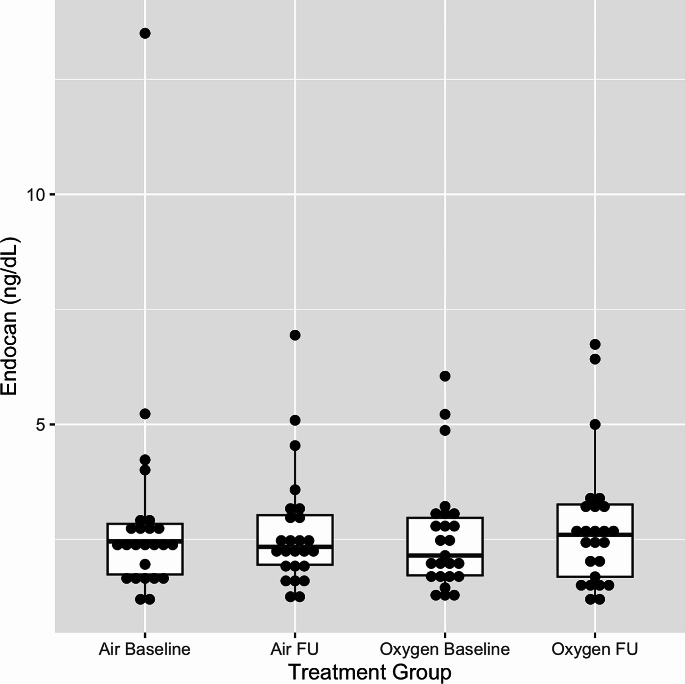
Combined boxplot and dotplot of endocan levels in both the air (sham) and the supplemental oxygen and arms at baseline and follow-up. Each individual data point is represented by a dot. The edge of the boxes represent the first and third quartiles with whiskers extending to 1.5 IQR from the median

Baseline endocan values were comparable in both arms with median endocan levels of 2.15 ng/ml (1.70, 3.00) in the sham arm and median levels of 2.46 ng/ml(1.74, 2.86) in the supplemental oxygen arm. In our mixed effect models, there was no significant effect of oxygen versus air on adjusted endocan levels at follow-up (treatment effect + 0.52 ng/ml, 95%CI -0.21 to + 1.25, *p* = 0.16). Assessing the two arms individually, there was no significant effect of CPAP withdrawal in the sham arm (treatment effect − 0.30 ng/ml, 95%CI -0.89 to + 0.30, *p* = 0.31) or in the supplemental oxygen arm (treatment effect + 0.22 ng/ml, 95%CI 0.00 to + 0.44, *p* = 0.05). Endocan levels at baseline and follow-up are shown for both arms in Fig. 1.

We conducted a sensitivity excluding the values with undetectable levels and one extreme outlier (endocan value 13.5 ng/ml). There remained no significant effect of supplemental oxygen versus sham on adjusted endocan levels at follow-up (treatment effect + 0.23 ng/ml, 95%CI -0.06 to + 0.51, *p* = 0.12). There remained no significant effect of CPAP withdrawal in the sham arm (treatment effect − 0.04 ng/ml, 95%CI -0.25 to + 0.18, *p* = 0.73) or in the supplemental oxygen arm (treatment effect + 0.22 ng/ml, 95%CI -0.02 to + 0.47, *p* = 0.07).

We conducted a sensitivity analysis only including participants with severe OSA at diagnosis (*n* = 16). There remained no significant effect of supplemental oxygen versus sham on adjusted endocan levels at follow-up (treatment effect + 0.70 ng/ml, 95%CI -0.33 to + 1.74, *p* = 0.17).

### Correlations between markers of intermittent hypoxia and endocan levels

We assessed the correlations between the change in endocan levels from baseline to follow-up to parameters of intermittent hypoxia in the supplementary oxygen and air arms separately, and the correlations between the difference in the change in endocan levels and the difference in markers of intermittent hypoxia, oxygen minus air. We found no significant correlations in either the oxygen or the air arms (Table 2).

**Table 2:** The correlation between change in endocan levels and measures of intermittent hypoxia (ODI, *%T90* and HB_OXI_) in the oxygen and air arms, and the correlation between the difference in the change in endocan levels and the change in measures of intermittent hypoxia (ODI, *%T90* and HB_OXI_). HB_OXI_ = OSA specific hypoxic burden as measured by oximetry, ODI: Oxygen desaturations index > 4%, %T90 = percentage of time with oxygen saturations less than 90%, 95%CI = 95% confidence interval

Oxygen arm			
	ODI (events/hour)	%T90 (%)	Hypoxic burden (%.min/hour)
Change in endocan levels	Rho=-0.12 *P* = 0.58	Rho=-0.16 *P* = 0.43	Rho=-0.31 *P* = 0.13
Air arm			
Change in endocan levels	Rho=-0.36 *P* = 0.07	Rho = 0.02 *P* = 0.91	Rho=-0.22 *P* = 0.30
Difference in levels, oxygen minus air			
Change in endocan levels	Rho=-0.35 *P* = 0.09	Rho=-0.05 *P* = 0.83	Rho=-0.33 *P* = 0.11

## Discussion

Our study shows that when using a sensitive assay, plasma endocan levels are detectable in the majority of patients with moderate to severe OSA, both before and after CPAP withdrawal. This contrasts with our previous findings where plasma endocan levels were undetectable in over 50% of patients with OSA. However, there was no effect of supplemental oxygen or CPAP withdrawal on endocan levels despite endocan being readily detected in our study.

Endocan levels are known to be elevated in OSA when comparing patients with OSA to matched controls [4]. A prospective study of patents with moderate to severe OSA, found that endocan levels correlated with extent of endothelial dysfunction as measured by flow mediated dilation and three months of CPAP therapy was associated with reductions in endocan levels [5]. We have shown however that 14 nights of CPAP withdrawal had no effect on plasma endocan levels, similar to our previous findings [6]. It is possible that 14 nights is not sufficient time for changes in endocan to be observed. Whilst 14 nights of CPAP withdrawal is sufficient to impair flow mediated dilatation which is a key marker of nitric oxide mediated endothelial function [10], it may be insufficient to lead to elevations in circulating endocan.

Intermittent hypoxia is a potential mechanism for the development of endothelial dysfunction and atherosclerosis in OSA [1]. However, intermittent hypoxia mediated endothelial damage is dependent on inflammation and oxidate stress [11], it is possible that 14 nights of CPAP withdrawal is not sufficient to cause systemic inflammation and oxidative stress [12]. Our results may have been influenced by 28% of participants being on statin therapy which is known to lower endocan levels [13], and which may have attenuated any effect of supplemental oxygen or CPAP withdrawal on endocan levels but which did not stop us from detecting circulating endocan.

In summary, we have shown that endocan levels are detectable within plasma in the majority of patients with OSA when using a more sensitive immunoassay. However, we found no effect of 14 nights of CPAP withdrawal either with or without supplemental oxygen on endocan levels. Supplemental oxygen is not currently recommended as a treatment option for OSA. Before it could be recommended, further work is needed to determine the longer-term effects of CPAP treatment and supplemental oxygen on cardiovascular physiology and its longer-term effects and safety.

## Data Availability

The data that support the findings of this study are not openly available but are available from the corresponding author upon reasonable request.
